# Scoliosis dance therapy: a worth-while addition to conservative scoliosis treatments? A pilot study evaluating the effect of a DVD led instruction on the wellbeing of scoliosis sufferers

**DOI:** 10.1186/1748-7161-7-S1-P24

**Published:** 2012-01-27

**Authors:** K Bauknecht

**Affiliations:** 1 Centre for Paediatric Physiotherapy, Neutraubling, Regensburg, Germany

## Background

Most teenagers with scoliosis suffer from spine deformation and from having to wear a brace. An unfavourable lower sagittal profile of scoliotic vertebrae with a lack of flexibility and a reduced equilibrium is often accompanied by a less developed body feeling. Supporting young people in correcting their posture with conventional treatment can be challenging. The importance of correcting deformities is often most difficult to get across to patients during puberty, resulting in irregular and insufficient exercise patterns.

## Aims

A new form of scoliosis treatment, giving up static exercising for a more dynamic, dance-like therapy, has been developed allowing a playful and self determined way of exercising. The objective of this pilot study was to gain a first impression on how this new form of treatment was received by young patients.

## Materials and methods

Twenty individuals were given a DVD showing a sequence of about 200 correcting movements to support them with their daily exercises. The effect on various aspects of their lives was subsequently evaluated via a structured questionnaire.

## Results

All participants noticed a positive effect on their body awareness and reported improved balance. Exercising took place more frequently and with more enthusiasm. The overall appearance seemed to have improved.

## Conclusions

Changing from static to dynamic scoliosis treatment had a positive effect on several factors and improved scoliosis therapy in this small group of patients. A prospective controlled study with a larger sample of patients has to take place before further conclusions can be drawn.

